# Performance of ChatGPT on the India Undergraduate Community Medicine Examination: Cross-Sectional Study

**DOI:** 10.2196/49964

**Published:** 2024-03-25

**Authors:** Aravind P Gandhi, Felista Karen Joesph, Vineeth Rajagopal, P Aparnavi, Sushma Katkuri, Sonal Dayama, Prakasini Satapathy, Mahalaqua Nazli Khatib, Shilpa Gaidhane, Quazi Syed Zahiruddin, Ashish Behera

**Affiliations:** 1 Department of Community Medicine All India Institute of Medical Sciences Nagpur, Maharashtra India; 2 Melmaruvathur Adhiparasakthi Institute of Medical Sciences and Research Melmaruvathur India; 3 Department of Community Medicine and School of Public Health Postgraduate Institute of Medical Education and Research Chandigarh India; 4 Department of Community Medicine KMCH Institute of Health Sciences and Research Coimbatore India; 5 Department of Community Medicine ESIC Medical College & Hospital, Sanathnagar Hyderabad India; 6 Center for Global Health Research Saveetha Medical College and Hospital Saveetha Institute of Medical and Technical Sciences, Saveetha University Chennai India; 7 Medical Laboratories Techniques Department AL-Mustaqbal University, Hillah Babil Iraq; 8 Division of Evidence Synthesis Global Consortium of Public Health and Research Datta Meghe Institute of Higher Education Wardha India; 9 Centre for One Health Education, Research & Development Jawaharlal Nehru Medical College Datta Meghe Institute of Higher Education Wardha India; 10 Global Health Academy Division of Evidence Synthesis School of Epidemiology and Public Health and Research, Jawaharlal Nehru Medical College Datta Meghe Institute of Higher Education and Research Wardha India; 11 Department of Internal Medicine Postgraduate Institute of Medical Education and Research Chandigarh India

**Keywords:** artificial intelligence, ChatGPT, community medicine, India, large language model, medical education, digitalization

## Abstract

**Background:**

Medical students may increasingly use large language models (LLMs) in their learning. ChatGPT is an LLM at the forefront of this new development in medical education with the capacity to respond to multidisciplinary questions.

**Objective:**

The aim of this study was to evaluate the ability of ChatGPT 3.5 to complete the Indian undergraduate medical examination in the subject of community medicine. We further compared ChatGPT scores with the scores obtained by the students.

**Methods:**

The study was conducted at a publicly funded medical college in Hyderabad, India. The study was based on the internal assessment examination conducted in January 2023 for students in the Bachelor of Medicine and Bachelor of Surgery Final Year–Part I program; the examination of focus included 40 questions (divided between two papers) from the community medicine subject syllabus. Each paper had three sections with different weightage of marks for each section: section one had two long essay–type questions worth 15 marks each, section two had 8 short essay–type questions worth 5 marks each, and section three had 10 short-answer questions worth 3 marks each. The same questions were administered as prompts to ChatGPT 3.5 and the responses were recorded. Apart from scoring ChatGPT responses, two independent evaluators explored the responses to each question to further analyze their quality with regard to three subdomains: relevancy, coherence, and completeness. Each question was scored in these subdomains on a Likert scale of 1-5. The average of the two evaluators was taken as the subdomain score of the question. The proportion of questions with a score 50% of the maximum score (5) in each subdomain was calculated.

**Results:**

ChatGPT 3.5 scored 72.3% on paper 1 and 61% on paper 2. The mean score of the 94 students was 43% on paper 1 and 45% on paper 2. The responses of ChatGPT 3.5 were also rated to be satisfactorily relevant, coherent, and complete for most of the questions (>80%).

**Conclusions:**

ChatGPT 3.5 appears to have substantial and sufficient knowledge to understand and answer the Indian medical undergraduate examination in the subject of community medicine. ChatGPT may be introduced to students to enable the self-directed learning of community medicine in pilot mode. However, faculty oversight will be required as ChatGPT is still in the initial stages of development, and thus its potential and reliability of medical content from the Indian context need to be further explored comprehensively.

## Introduction

Artificial intelligence (AI), generally defined as the simulation of human intelligence by a machine, was developed in the late 20th century. ChatGPT, which debuted a preview version in November 2020, is a new entrant in the AI field with enhanced, user-friendly, and near human–like attributes [1,2]. ChatGPT belongs to the category of large language models (LLMs) as a deep-learning AI trained by imbibing large volumes of texts to produce human-like outcomes. ChatGPT is the successive version of GPT-3 and draws 96 million monthly visitors [3], including 13 million unique visitors daily during January 2023 [4], with more than 100 million users [3] registered within the short span of its launch. More recently, the search giant Google released their LLM-based chatbot “Gemini” (formerly known as “Bard”) to the public [5].

The challenges of medical education [6] and the perceived ineffectiveness of traditional teaching methods such as lectures [7] are obstacles to the effective learning of medical students for which technology poses as a solution [6]. Community medicine—a vital subject taught over the 3 out of 4 years of medical school, with an additional 3 months of internship training—deals with public health topics in the undergraduate medical curriculum in India [8]. The community medicine curriculum plays a crucial role in training medical students to understand the community’s public health needs and develop the necessary skills to promote health and prevent disease [9].

AI-assisted learning and teaching practices are already widely used [10]. LLMs have also been integrated into allopathic and alternate systems of medical education through various aspects such as by solving multiple-choice questions with reasoning, answering queries, providing interactive practice cases, and facilitating differential diagnosis [11-13]. The potential scope for LLMs in medical education also includes curriculum development, personalized study plans, and program evaluation and monitoring [14]. ChatGPT’s inherent property of the “transformer model” has been reported to assist in writing review articles on health [15]. The interactive digital interface and prompt-based responses of GPT models with access to a multidomain database [16] offer feasibility for students to use these tools during the “interested learner” stage of self-directed learning (SDL) [15], which is an essential principle of adult learning.

ChatGPT has been shown to complete exams from varied domains such as business administration [17], the medical licensing exam for medical graduates [18], and the parasitology exam at the undergraduate medical level [19], with mixed results. ChatGPT’s ability to answer and explain the questions administered to medical students in their evaluations indicate its potential role as a learning assistant to students. In due course, students may use multidisciplinary LLMs such as ChatGPT in their learning. This is particularly relevant for a subject such as community medicine, which requires real-world application of knowledge beyond medicine to multidisciplinary determinants of health. However, when such tools are used, it is essential that the responses given are relevant and accurate. Thus, assessing the LLM’s capacity to accurately answer and explain the community medicine examination questions and concepts administered to students is imperative. Furthermore, such evaluations will provide a better understanding of how language models can be used in medical education and the challenges that must be overcome to fully realize their potential. In this background, the primary aim of this study was to validate the ability of ChatGPT to answer the undergraduate medical community medicine examination. We also compared the scores obtained on the examination by ChatGPT with those obtained by the students.

## Methods

### Design, Setting, and Population

This was a retrospective study based on secondary data conducted in February 2023 at a publicly funded medical college in Hyderabad, India. There are approximately 450 medical undergraduate students currently being trained at the institute. We enrolled all 94 students in the Bachelor of Medicine and Bachelor of Surgery (MBBS) Final Year–Part I curriculum. The Final Year–Part I program comprises three subjects: otorhinolaryngology, ophthalmology, and community medicine. Students are taught and trained in practical and theoretical aspects in these subjects for a period of 12 months during the course of their MBBS studies.

### Study Tool

Two question papers from the syllabus of community medicine for the MBBS requirements were set with a total of 100 marks (India uses a “mark” scoring system to rate exam answers, with marks having a similar meaning as points) distributed among 40 questions in two papers with 20 questions each. There are three sections in both papers, with different weightage of marks for each section: section 1 had 2 long essay–type questions worth 15 marks each, section 2 had 8 short essay–type questions worth 5 marks each, and section 3 had 10 short-answer questions worth 3 marks each.

### Evaluation of ChatGPT Responses

The same questions from the internal assessment exam for the students conducted by the Department of Community Medicine, ESIC Medical College and Hospital, Hyderabad in January 2023 were input as prompts to ChatGPT by one author (APG) on January 25, 2023. The author has a registered open-access account with OpenAI, which has released ChatGPT. 

The answers given by ChatGPT 3.5 to each question were recorded and evaluated by three evaluators who had previously been involved in assessing the answer sheets of the students. The internal examination paper of the students is assessed by faculty at both the Assistant Professor and Professor levels; hence, we included two evaluators of different cadres to eliminate potential bias between a senior (SK) and junior (SD) faculty member in their evaluations owing to differences in experience. Despite this difference in experience, both evaluators have the same educational background (MD in community medicine). Adhering to the double-evaluation guidelines of the university to which the institute is affiliated, if there was a difference in marks of more than 33% awarded to a question by the two evaluators, a third evaluator (APG; assistant professor, MD in community medicine) was called in and their evaluation mark was considered to be the final score for that question.

### Qualitative Evaluation of ChatGPT Responses

Apart from scoring for the content, the evaluators assessed the responses to each question under three subdomains to further analyze the quality of ChatGPT responses: relevancy (“Is the answer relevant to the prompt?”), coherence (“Is the description in the answer internally coherent?”), and completeness (“Does the answer sufficiently address all parts of the prompt?”). Each question was scored in these subdomains on a Likert scale of 1-5. The average of the two evaluators was taken as the subdomain score of the question.

### Comparison Between ChatGPT and Student Scores

ChatGPT scores were compared with the average score of the Final Year–Part I students obtained during the internal examination. To calculate the average score of the students, their deidentified internal examination scores were obtained from the existing departmental registers. A score of ≥50% is considered the minimum passing percentage for the community medicine subject of the MBBS Final Year–Part I requirements.

### Data Analysis

Data analysis was performed in Microsoft Excel. The scores obtained by ChatGPT are expressed as percentages. The overall mean score of the MBBS students was calculated by adding the individual scores of all students and dividing by the number of students who had attended the internal assessment examination. Feedback from the evaluators (SK and SD) was obtained to explore their perception toward the ChatGPT responses. 

### Ethical Considerations

As this study was based on the use of secondary data without identifiers, on application (reference 799/U/IEC/ESICMC/F522/03/2023) to the Institutional Ethics Committee of ESIC Medical College and Hospital, Hyderabad, the study was determined to be exempted from ethical review.

## Results

### Scores of Students and ChatGPT Responses

**Figure 1:**
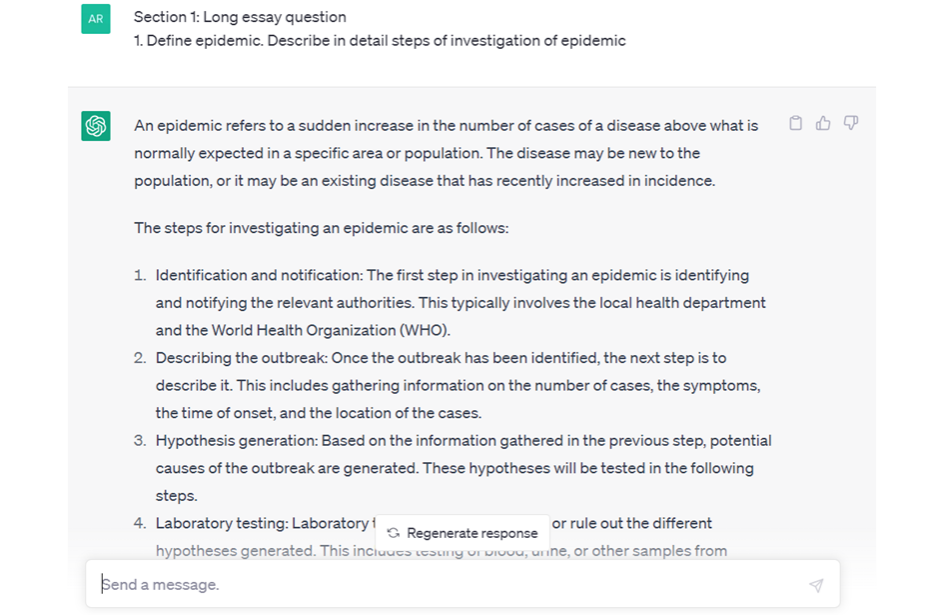
Sample ChatGPT response to a community medicine examination question.

The student responses were scored separately and then the prompts to ChatGPT were delivered. The average score of the students was 43% (43/100) on paper 1 and 45% (45/100) on paper 2 (Multimedia Appendix 1). A sample response given by ChatGPT is shown in Figure 1. Complete answers given by ChatGPT, along with the prompts given by the investigators, for all questions are provided in Multimedia Appendices 2 and 3.

The mean score of the responses for the internal assessment community medicine exam questions by ChatGPT was 133 (66.7%); ChatGPT scored 72% (72/100) on paper 1 and 61% (61/100) on paper 2.

### Qualitative Evaluation of ChatGPT Responses

In terms of the relevancy of the responses given by ChatGPT, 35 out of 40 questions (88%) were rated to have more than 50% of the maximum score (more than 3 out of 5), while 36 of the 40 questions (90%) had a coherence score greater than 50% of the maximum score. Regarding completeness, ChatGPT’s responses to 32 of the 40 questions (80%) were rated to have more than 50% of the maximum score (Multimedia Appendix 1).

Qualitative feedback was obtained from the evaluators regarding the responses to the questions given by ChatGPT. Medical and health technical terminologies were used relatively less frequently by ChatGPT in comparison with the student responses. Figurative representations (images/diagram) were lacking in the free version of ChatGPT 3.5. This is an inherent limitation of the tool since ChatGPT cannot generate images. ChatGPT did not split the long paragraphs into subheadings, which was implemented in some of the student responses as a strategy to ease understanding. Since the subject was primarily focused on the Indian context, the evaluators also opined that ChatGPT did not cover this context adequately in the responses. The more Western-oriented responses might be because ChatGPT is mainly trained based on English content available on the internet. Supporting this hypothesis, OpenAI has also noted that the role of ChatGPT in a classroom setting is currently biased toward Western settings, while non-Western perspectives are inequitably incorporated within the model [20]. The scope for expansion of the perspectives beyond borders is possible in the future.

The responses to the epidemiological questions need to be elaborately discussed with more clarity using examples and illustrations. Regarding long essay–type descriptive answers, ChatGPT did not go beyond the length limit of 1000-1200 words, even after the prompt was fine-tuned to specify to answer the questions in at least 3000 words. ChatGPT responded negatively when asked whether it has any word limits for a single response. However, the issue of a smaller number of words also persisted in subsequent attempts.

## Discussion

### Principal Findings

In this study, ChatGPT scored 72.3% on paper 1 and 61% on paper 2, while the mean scores of the students were 43% and 45%, respectively. The responses of ChatGPT were also rated to be satisfactorily relevant (88%), coherent (90%), and complete (80%) for most of the questions. Therefore, ChatGPT passed the Indian undergraduate-level community medicine exam with an overall score of 66.7%, which is above the minimum pass criterion for students under the university norms (50%), and hence can be considered satisfactory.

Smarter and faster AI tools have been massively introduced in academia and health care during the 21st century. Almost all disciplines worldwide harness AI’s rapidly emerging principles and applications. Medical education can also leverage the potential of AI in providing engaged and self-directed learning for students [16,21]. This efficacy might be due to the extensive training database of ChatGPT, which comprised 300 billion words spanning multiple disciplines and the “transformer model.” Earlier learning models were restricted to a specific task (ie, narrow AI). After the “transformer model” was introduced in 2017, a more comprehensive, cross-learning tool using a transfer-learning strategy emerged. Transfer learning enables the tool/software to apply the learnings from one task to execute another [22].

Previous studies have shown mixed results regarding ChatGPT’s ability to answer questions for various subjects. ChatGPT cleared the Master of Business Administration (MBA) examination with a B to B– grade at the University of Pennsylvania, administered under research mode by the business school professor [17]. The multiple-choice question–based test in family medicine in Belgium was cleared by ChatGPT, with better performance found on the negatively worded questions compared to that of the students [23]. Other studies have demonstrated the at or near passing ability of ChatGPT in cracking the 3-step United States Medical Licensing Exam (USMLE) [18,24]. Although ChatGPT’s ability to answer parasitology questions was not comparable to that of the medical students of Korea, the responses provided by ChatGPT had an acceptable explanation for the questions [19]. In contrast, in this study, we found that ChatGPT surpassed the mean score of the students. This might be due to the varied methodology applied compared to that adopted in previous studies. The Korean study was specific to the field of parasitology, and the medical students were administered the exam immediately after completing the module. In contrast, in this study, students were answering questions related to the entire syllabus of community medicine that they had been learning for 3 years. In the current setting, the internal examinations only serve as trials for the university examinations and do not determine the student’s qualification per se. Hence, the motivation factors also vary. Thus, the quantum of the syllabus, timing of the examination, and motivations might have contributed to the students’ relatively low scores in the current setting. The median time for the students to answer each paper (20 questions) was 3 hours, whereas ChatGPT provided faster responses (5-10 minutes for each paper).

GPT-4, the latest version of ChatGPT, has scored more than 80% in all 3 steps of the USMLE [25] and demonstrated an accuracy rate of 76.4% in understanding and answering the surgery board exam questions from Korea [26]. The Korea study also observed a significant difference in the accuracy between the original version (3.5) and the advanced version (4.0) of ChatGPT, indicating constant enhancement of the tool [26]. A study from Australia reported that despite the ability of GPT-4 to give a structured and comprehensive response to the frequently asked questions in breast augmentation procedures in the plastic surgery specialty, it could not provide tailored advice. In some cases, the responses were even found to be inappropriate [27]. Although ChatGPT did not reach the passing threshold in the life support exams (basic and advanced) conducted by the American Heart Association, it was reported to have the potential to enable SDL by assisting the students in preparing for the exams [28]. The real-time feedback provided by ChatGPT can also enable tailoring and fine-tuning the learning and teaching strategies [29]. A study from Pittsburgh in the United States reported mixed findings regarding the capacity of ChatGPT in answering the questions on the plastic surgery in-service examination [30]. While ChatGPT was found to provide answers that were on par with those of the first-year plastic surgery service residents, it could not match the performance of residents with advanced years of training [30].

The responses of ChatGPT in this study were considered to be satisfactorily relevant, coherent, and complete for most of the questions (>80%). Previous studies have reported relatively more relevant, accurate, and congruent answers being provided by ChatGPT than the earlier AI systems [24,28]. Given the better performance of ChatGPT on the student exams, aspersions on the existing evaluation techniques of medical students have been cast [31]. The need to reevaluate the existing assessment tools with more critical thinking–based training and testing, along with less memory-based evaluation, has also been proposed [31]. ChatGPT-assisted framing of questions and clinical scenarios may also be undertaken for student assessments, thus aiding faculty [21]. The phenomenon of hallucination, wherein ChatGPT provided a wrong answer but with a confident explanation, has also been reported in the literature [23].

### Strengths and Limitations

This study represents an early attempt to systematically evaluate ChatGPT’s capacity to answer examination questions on an undergraduate subject covering public health concepts, involving two independent subject experts for assessment. We further evaluated the responses regarding relevancy, coherence, and completeness. However, this study was not without limitations. To maintain uniformity and avoid subjectivity, we adopted a similar prompt system for the students and ChatGPT while administering the paper. However, ChatGPT requires more specific and detailed prompts than the students since students are focused on a specific domain, whereas ChatGPT is a multidisciplinary tool. The ability of ChatGPT in terms of word limits is also ambiguous. This might have restricted ChatGPT from exceeding certain word limits, affecting its ability to provide an adequately descriptive response. At the time of the study, information in the ChatGPT database had been updated up to 2021; hence, the tool could not answer or understand the scope of the latest advances in public health. In addition, it was not feasible to blind the evaluators to ensure they remained unbiased to the responses of students and AI. This bias could be in either direction (favorable or against ChatGPT), according to the subjective opinion of the evaluators. We tried to adjust for this potential bias by having two independent evaluators. Moreover, the comparison was based on a single internal assessment conducted among students from a single institute, which limits the generalizability of the findings. Ethical issues surrounding the use of these LLM tools in medical and health care workers’ education, especially in terms of students using the tool to generate assignments, also remain to be considered while undertaking related research and implementation [32,33].

### Conclusion

In conclusion, ChatGPT effectively answered questions related to the Indian undergraduate-level community medicine curriculum. ChatGPT demonstrated adequate comprehension, relevancy, and correctness in answering the community medicine–related questions, indicating its potential as an assistive tool in medical education in India. However, the need for continuous improvement in accuracy and the challenges associated with a contextual understanding of ChatGPT must also be considered. Additionally, efforts should be made to address the potential biases and limitations of language models, ensuring their ethical and responsible use in educational settings. ChatGPT may be introduced to students to enable the SDL of community medicine in a pilot mode under faculty oversight, as its development remains in the initial stages, while the potential and reliability of medical content from the Indian context need to be more comprehensively explored. Integrating ChatGPT as a complementary tool in the learning process could contribute to a more novel, interactive, and engaging educational experience for medical students, ultimately enhancing their understanding and application of community medicine principles. Operational studies exploring the feasibility, experience, and effectiveness of LLMs such as ChatGPT in the actual learning of Indian medical students should be undertaken in the future.

## Appendix

Multimedia Appendix 1 Evaluation sheet of ChatGPT responses.

Multimedia Appendix 2 ChatGPT answers for the questions in paper 1.

Multimedia Appendix 3 ChatGPT answers for the questions in paper 2.

## Abbreviations

AI: artificial intelligence
LLM: large language model
MBA: Master of Business Administration
MBBS: Bachelor of Medicine and Bachelor of Surgery
SDL: self-directed learning
USMLE: United States Medical Licensing Exam

## Data Availability

All data generated or analyzed during this study are included in this published article and supplementary files.
