# Supplementary material for: Performance of ChatGPT on the India Undergraduate Community Medicine Examination: Cross-Sectional Study
Source: JMIR Form Res. 2024 Mar 25;8:e49964. doi: 10.2196/49964 (PMC11002731; doi:10.2196/49964)
Supplement: Multimedia Appendix 2 [file formative_v8i1e49964_app2.pdf]

Multimedia Appendix 2: ChatGPT answers for the questions in Paper I

Section 1: Long essay question

1. Define epidemic. Describe in detail steps of investigation of epidemic

An epidemic refers to a sudden increase in the number of cases of a disease above what is normally expected in a specific area or population. The disease may be new to the population, or it may be an existing disease that has recently increased in incidence.

The steps for investigating an epidemic are as follows:

1. Identification and notification: The first step in investigating an epidemic is identifying and notifying the relevant authorities. This typically involves the local health department and the World Health Organization (WHO).
2. Describing the outbreak: Once the outbreak has been identified, the next step is to describe it. This includes gathering information on the number of cases, the symptoms, the time of onset, and the location of the cases.
3. Hypothesis generation: Based on the information gathered in the previous step, potential causes of the outbreak are generated. These hypotheses will be tested in the following steps.
4. Laboratory testing: Laboratory testing is used to confirm or rule out the different hypotheses generated. This includes testing of blood, urine, or other samples from infected individuals.
5. Field investigation: A field investigation is conducted to gather additional information on the outbreak. This includes conducting interviews with affected individuals, visiting the places where the cases occurred, and collecting environmental samples.
6. Data analysis: The data collected in the previous steps is analyzed to determine the cause of the outbreak. This includes looking for patterns in the data, such as clusters of cases, and identifying any common exposures among affected individuals.
7. Control and prevention: Once the cause of the outbreak has been determined, control and prevention measures can be implemented to stop the spread of the disease and prevent future outbreaks. This may include measures such as vaccination, quarantine, and treatment of affected individuals.
8. Reporting: The final step in the investigation of an epidemic is reporting the findings to the relevant authorities and the public. This includes a detailed report on the cause of the outbreak and the control and prevention measures that were taken.

2. Describe in detail the different methods of disposal of waste

Waste disposal refers to the process of getting rid of waste materials in a safe and environmentally friendly manner. There are several different methods of waste disposal, each with its own advantages and disadvantages.

1. Landfilling: Landfilling is the most common method of waste disposal. It involves burying waste in a designated area. This method is relatively cheap and easy to

implement, but it can have negative environmental impacts, such as groundwater contamination and the release of methane gas.

2. Incineration: Incineration involves burning waste at high temperatures. This method is effective at reducing the volume of waste and can produce energy in the form of heat or electricity. However, it can also produce harmful pollutants such as dioxins and heavy metals, and the ash produced must be disposed of safely.
3. Composting: Composting is the process of breaking down organic waste materials, such as food scraps and yard waste, into a nutrient-rich soil amendment. This method is environmentally friendly and can produce a valuable product, but it requires a significant amount of space and can be time-consuming.
4. Recycling: Recycling involves the collection, separation, and processing of materials for reuse. This method is beneficial for the environment as it reduces the need for new materials, but it can be costly to implement and may not be feasible for all types of waste.
5. Anaerobic Digestion: is a process that converts organic waste into biogas and a nutrient-rich fertilizer. This method is environmentally friendly and can produce energy and fertilizer, but it requires specific conditions and can be costly to implement.
6. Plasma Gasification: is a process that converts waste into a gas which can be used for energy production. It is considered as a clean and advanced technology for waste management, but it requires high-temperature and high-pressure condition, which makes it expensive to implement.

The most appropriate method of waste disposal will depend on the type and volume of waste, as well as the resources and regulations in place in a particular area. It is important to consider the environmental impacts and costs of each method before making a decision on the best method of disposal.

Section 2: Short essay

3. Effects of Heat stress

Heat stress is a condition that occurs when the body is unable to cool itself properly, leading to an increase in body temperature. The effects of heat stress can range from mild to severe and can include a variety of symptoms.

1. Heat cramps: Heat cramps are muscle pains or spasms that occur due to dehydration and electrolyte imbalances. They are a common symptom of heat stress and can occur in the legs, arms, or stomach.
2. Heat exhaustion: Heat exhaustion is a more severe form of heat stress and is characterized by symptoms such as heavy sweating, weakness, dizziness, and fainting. It can occur as a result of prolonged exposure to high temperatures or dehydration.
3. Heat stroke: Heat stroke is a medical emergency and is the most severe form of heat stress. It occurs when the body's temperature regulation system fails, leading to a body temperature of 104 degrees Fahrenheit or higher. Symptoms include confusion, seizures, and loss of consciousness.

4. Cardiovascular effects: Heat stress can also have an effect on the cardiovascular system, leading to increased blood pressure, heart rate, and strain on the heart.
5. Fatigue: Heat stress can cause fatigue and reduction in work efficiency.
6. Skin problems: Heat stress can cause skin problems such as rashes, sunburn, and heat rash.
7. Respiratory problems: Heat stress can cause respiratory problems such as asthma, bronchitis and make them worse.

Heat stress can be prevented by staying indoors in air-conditioned places during hot weather, drinking plenty of fluids, wearing loose and lightweight clothing, and taking regular breaks in the shade or in a cool area. If you suspect heat stress, it is important to seek medical attention immediately, especially for heat stroke.

4. Epidemic dropsy

Epidemic dropsy is a disease that is caused by the accumulation of fluid in the body's tissues. It is also known as edema, and it is characterized by swelling, particularly in the legs, ankles, and feet. The condition is more common in women, and it is most frequently seen in the elderly and those with chronic illnesses.

Epidemic dropsy is caused by the disruption of the body's normal fluid balance. This can be due to a variety of factors, including heart failure, kidney disease, and certain medications. In some cases, the cause of the condition is unknown.

The symptoms of epidemic dropsy include swelling of the legs, ankles, and feet, as well as tightness and a feeling of heaviness in the affected area. The skin may also appear shiny and stretched. In severe cases, the swelling can cause pain and difficulty walking.

The diagnosis of epidemic dropsy is based on a physical examination and medical history. A blood test may also be done to check for underlying causes of the condition.

Treatment of epidemic dropsy depends on the underlying cause. In cases where the cause is unknown, treatment is focused on relieving symptoms and preventing complications. This may include the use of diuretics to reduce the amount of fluid in the body, compression stockings to reduce swelling, and elevation of the affected limb. In cases where the condition is caused by an underlying medical condition, treatment is directed at addressing that condition.

Prevention of epidemic dropsy includes maintaining a healthy lifestyle, such as eating a balanced diet and regular exercise, controlling hypertension and diabetes, and avoiding standing or sitting in one place for extended periods.

It's important to note that if you experience symptoms of epidemic dropsy, it is important to seek medical attention as it may be indicative of an underlying condition that needs to be addressed.

5. PQLI

The Physical Quality of Life Index (PQLI) is a composite measure of a country's overall well-being, based on three underlying indicators: infant mortality rate, literacy rate, and life expectancy at age one. The PQLI was developed by Mahbub ul Haq, a Pakistani economist in the 1970s.

The PQLI is calculated by taking the average of the three underlying indicators, each of which is given equal weight. The infant mortality rate is taken as a measure of a country's health care system, the literacy rate is used as a measure of education, and life expectancy at age one is used as a measure of overall well-being. A higher PQLI score indicates a higher level of well-being.

The PQLI has been widely used as a measure of a country's development, particularly in the 1970s and 1980s. It is considered to be a simple and easily understood measure of a country's overall well-being. However, it has some limitations as well. It does not take into account other important factors such as income, poverty, and access to clean water and sanitation, which are also important indicators of well-being.

Despite its limitations, the PQLI is still widely used as an easy-to-understand measure of a country's overall well-being. It is used by organizations such as the United Nations Development Programme (UNDP) to track progress towards the Millennium Development Goals. It is also used by governments, researchers, and other organizations as a tool for monitoring and evaluating development programs and policies.

In summary, PQLI is a composite measure of a country's overall well-being, based on three underlying indicators: infant mortality rate, literacy rate, and life expectancy at age one. It was widely used as a measure of a country's development in the past but has some limitations and other factors such as income, poverty, and access to clean water and sanitation are also important for a comprehensive understanding of a country's well-being.

6. Social factors influencing health of the people

Social factors play a significant role in shaping an individual's health. These factors include but are not limited to:

1. Socioeconomic status: Socioeconomic status, including income, education, and occupation, is strongly associated with health outcomes. People with lower socioeconomic status tend to have worse health outcomes than those with higher socioeconomic status.
2. Housing: Adequate and safe housing is important for good health. People who live in overcrowded or substandard housing conditions are more likely to suffer from poor health.
3. Social support: Having a strong network of family, friends, and community support can help improve an individual's health outcomes.
4. Discrimination: Discrimination and social exclusion can have a negative impact on an individual's health. People who experience discrimination based on race, ethnicity,

gender, sexual orientation, and other factors are more likely to have worse health outcomes.
5. Education: Education is an important social determinant of health. People who have higher levels of education tend to have better health outcomes than those who have less education.
6. Employment: Unemployment, underemployment, and job insecurity are associated with poor health outcomes.
7. Access to health care: Access to affordable and quality health care is important for good health. People who have limited access to health care are more likely to have worse health outcomes.
8. Food security: Access to healthy and affordable food is important for good health. People who live in food-insecure households are more likely to suffer from poor health.
9. Neighborhood conditions: The conditions of the neighborhood in which people live can have a significant impact on their health. People who live in neighborhoods with high crime rates, poor air quality, and limited access to healthy food and recreational facilities are more likely to have worse health outcomes.

Overall, social factors can greatly influence the health of individuals and communities. Addressing these social determinants of health can help improve health outcomes and reduce health disparities.

7. Measures of dispersion

Measures of dispersion are used to describe how spread out a set of data is. There are several commonly used measures of dispersion, including:

1. Range: The range is the difference between the highest and lowest values in a set of data. It is the simplest measure of dispersion, but it is affected by outliers and is not always a reliable indicator of the overall spread of the data.
2. Variance: Variance is a measure of the average of the squared differences between each data point and the mean. It is commonly used in statistics, but it is affected by outliers and is not always easy to interpret.
3. Standard deviation: Standard deviation is a measure of the average deviation of each data point from the mean. It is calculated by taking the square root of the variance. It is a commonly used measure of dispersion and is often used in combination with the mean to describe a data set.
4. Interquartile Range (IQR): The interquartile range (IQR) is the difference between the 75th percentile and the 25th percentile of a data set. It is a measure of dispersion that is not affected by outliers and is often used to describe the spread of continuous data.
5. Coefficient of Variation (CV): The coefficient of variation (CV) is the ratio of the standard deviation to the mean, expressed as a percentage. It is used to compare the dispersion of different data sets, regardless of their means or units of measure.
6. Z-scores: Z-scores are standard scores that indicate how many standard deviations a data point is from the mean. It is used to identify outliers and to compare the relative position of a data point within a data set.

In summary, measures of dispersion are used to describe how spread out a set of data is. Some commonly used measures of dispersion include range, variance, standard deviation, interquartile range, coefficient of variation, Z-scores. Each of these measures has its own advantages and disadvantages, and the appropriate measure of dispersion will depend on the specific data set and the research question being addressed.

8. Cold chain

The cold chain refers to the system of maintaining a certain temperature range for perishable goods, such as vaccines, medicines, and food, during storage, transport and distribution. The purpose of a cold chain is to preserve the quality, safety, and effectiveness of these goods by keeping them at the appropriate temperature.

A cold chain typically consists of several stages, including:

1. Production: The first stage of the cold chain is the production of the perishable goods. This typically involves the manufacturing or packaging of the goods at a facility that is equipped to maintain the appropriate temperature range.
2. Storage: The next stage of the cold chain is storage. This includes the storage of the goods at a warehouse or distribution center that is equipped to maintain the appropriate temperature range.
3. Transportation: After the goods have been stored, they are transported to the next stage of the cold chain. This typically involves the use of refrigerated vehicles or containers to ensure that the goods are maintained at the appropriate temperature during transport.
4. Distribution: The final stage of the cold chain is distribution. This includes the delivery of the goods to the final destination, such as a hospital, clinic, or retail store. The goods may be stored at the final destination for a short period of time before being sold or distributed to the end user.

Proper cold chain management is essential to ensure that the perishable goods maintain their quality, safety, and effectiveness. It involves monitoring and controlling the temperature at each stage of the cold chain, and maintaining records of the temperatures throughout the process.

It's important to note that a break in the cold chain, such as a power failure or equipment malfunction, can result in the perishable goods being exposed to temperatures that are too high or too low, which can compromise their quality, safety, and effectiveness. Therefore, it's important to have backup systems in place to ensure that the cold chain can be maintained even in the event of a failure.

9. Health hazards of health care waste

Health care waste refers to any waste generated during the provision of health care services, including waste from hospitals, clinics, and other health care facilities. This type of waste can pose significant health hazards if it is not properly managed.

1. Infection: One of the main health hazards of health care waste is the risk of infection. This is because health care waste can contain infectious agents, such as bacteria, viruses, and other pathogens, that can cause serious illnesses if they come into contact with people.
2. Injury: Health care waste can also pose a risk of injury. This is because it can include sharp objects, such as needles and blades, that can cause cuts and puncture wounds.
3. Exposure to hazardous chemicals: Some of the chemicals used in health care, such as x-ray developing solutions, may be classified as hazardous waste and can be harmful to human health if not properly handled.
4. Pollution: Improper disposal of health care waste can also lead to pollution of the environment. This can have a negative impact on the health of people who live and work in the area, as well as on the health of animals and plants.
5. Fire hazards: Health care waste can be a fire hazard if not properly stored or disposed of. This is because it can include flammable materials, such as alcohol-based disinfectants, that can catch fire easily.
6. Cancer: Some of the waste material generated by health care facilities such as radioactive waste, genotoxic and carcinogenic chemicals, and cytotoxic drugs may cause cancer if not properly

10. Disaster preparedness

Disaster preparedness refers to the steps taken by individuals, organizations, and governments to plan for and respond to natural and man-made disasters. It is an ongoing process that involves assessing risks, developing plans and procedures, training personnel, and maintaining readiness.

1. Risk assessment: The first step in disaster preparedness is to conduct a risk assessment to identify the types of disasters that are most likely to occur in a given area and the potential impacts of those disasters. This information is used to develop plans and procedures to mitigate the impacts of the disaster.
2. Planning and Procedures: Once the risks have been identified, organizations and governments develop plans and procedures to respond to a disaster. This includes evacuation plans, emergency communication protocols, and procedures for providing emergency services such as medical care and shelter.
3. Training: Disaster preparedness also involves training personnel in emergency response, first aid, and emergency management procedures. This includes training for emergency responders, healthcare workers, and other personnel who will be involved in the response to a disaster.
4. Maintaining readiness: Disaster preparedness is an ongoing process and it's important to maintain readiness by regularly testing and updating plans and procedures, training personnel, and conducting drills and exercises to ensure that everyone is prepared to respond to a disaster.
5. Community engagement: Community engagement is an important aspect of disaster preparedness. This includes educating the community about disaster risks and providing information on how to prepare for, respond to, and recover from a disaster.

6. Coordination: Coordination between different organizations and agencies is essential for effective disaster preparedness and response. This includes coordination between government agencies, non-governmental organizations, and the private sector.

Disaster preparedness is a crucial step in protecting people, property, and the environment from the impacts of disasters. By assessing risks, developing plans and procedures, training personnel, and maintaining readiness, organizations and governments

Section 3: Short answers

11. Spectrum of health

The spectrum of health refers to the range of health states that individuals can experience, from optimal health to poor health. It encompasses physical, mental, and social well-being, and considers the presence or absence of disease or injury. The spectrum of health includes different levels, such as wellness, illness, disability, and death.

12. Differences between case control and cohort study

Case-control studies and cohort studies are two types of observational research designs that are used to investigate the relationship between a specific exposure and a particular outcome.

1. Case-control studies: A case-control study is a type of observational study that compares a group of individuals with a specific disease or condition (cases) to a group of individuals without the disease or condition (controls). The researcher then looks back in time to compare the exposure history of the cases and controls.
2. Cohort studies: A cohort study is a type of observational study that follows a specific group of individuals over time to assess their exposure to a particular risk factor and their subsequent development of a particular disease or condition. The researcher looks forward in time and follows a group of people who are exposed to a specific risk factor and a group of people who are not exposed.
3. Time of data collection: Case-control studies collect data retrospectively, after the outcome has occurred, while cohort studies collect data prospectively, before the outcome has occurred.
4. Sample size: Case-control studies typically have smaller sample sizes than cohort studies.
5. Data analysis: The data is analyzed differently in case-control and cohort studies. In case-control studies, the odds ratio is calculated to measure the association between the exposure and the outcome, while in cohort studies, the relative risk is calculated.

In summary, case-control studies and cohort studies are two types of observational research designs that are used to investigate the relationship between a specific exposure and a particular outcome. Case-control studies are retrospective, while cohort studies are prospective. They have different sample sizes and the data

13. Acculturation

Acculturation refers to the process by which individuals or groups of people adopt the customs, values, and behaviors of a new culture. This can occur as a result of migration, colonization, or other forms of cultural contact. Acculturation can have both positive and negative effects on an individual's or group's physical, mental, and social well-being.

Acculturation can be a complex and dynamic process that can involve changes in language, diet, clothing, social norms, and other aspects of culture. It can also lead to feelings of loss, identity conflict, and discrimination.

Acculturation can be measured through various methods like self-report questionnaires, interviews, observation and others. Some of the most common methods include the Suinn-Lew Asian Self-Identity Acculturation Scale, the Acculturation Rating Scale for Mexican Americans, and the Short Acculturation Scale for Hispanics.

Overall, acculturation is a process of cultural change that can have a significant impact on the lives of individuals and groups. It can lead to positive changes such as increased economic opportunities, but it can also lead to negative effects such as discrimination and loss of cultural identity.

14. Effects of noise pollution

Noise pollution refers to excessive, unwanted or disturbing sound that can have negative impacts on human health and well-being. The effects of noise pollution can be both physical and psychological.

1. Physical effects: Noise pollution can cause a range of physical effects, including hearing loss, high blood pressure, sleep disturbance, and cardiovascular disease.
2. Psychological effects: Noise pollution can also have negative psychological effects, such as stress, anxiety, and depression. It can also lead to decreased productivity, difficulty concentrating and memory problems.
3. Interference with communication: Noise pollution can interfere with communication, making it difficult for people to hear each other and understand what is being said.
4. Loss of biodiversity: Noise pollution can also have negative impacts on wildlife, disturbing their breeding, feeding, and migration patterns.
5. Effects on wildlife: Noise pollution can also have negative impacts on wildlife, disturbing their breeding, feeding, and migration patterns.
6. Effects on community: Noise pollution can also have negative effects on communities, leading to decreased property values and reduced quality of life.

Overall, noise pollution can have a wide range of negative effects on human health and well-being, as well as on the environment. It's important for individuals and communities to be aware of the sources of noise pollution and take steps to reduce exposure to excessive noise.

15. Eugenics

Eugenics is a social movement and set of theories that advocate for the improvement of human genetic traits through selective breeding and other forms of intervention. The movement emerged in the late 19th century and gained popularity in the early 20th century. Eugenics theories and practices have been used to justify various forms of discrimination and human rights abuses, such as forced sterilization and genocide.

1. Positive Eugenics: The goal of Positive Eugenics is to increase the number of people with desirable genetic traits. This can be achieved through methods such as encouraging the reproduction of people with desirable traits.
2. Negative Eugenics: The goal of Negative Eugenics is to decrease the number of people with undesirable genetic traits. This can be achieved through methods such as forced sterilization, abortion, and euthanasia of people with undesirable traits.
3. Forced Sterilization: Forced sterilization refers to the practice of surgically preventing individuals from being able to reproduce. This practice was used as a method of eugenics in many countries, including the United States, Canada, and Sweden, and was often directed at marginalized groups such as people with disabilities and ethnic minorities.
4. Eugenics and Race: Eugenics theories have been used to justify racism and discrimination against marginalized groups, such as people of color, immigrants, and people with disabilities.
5. Discredited theory: Eugenics is now widely discredited as a science and a social movement, and is widely considered to be a form of pseudoscience.

Eugenics is an unethical and discriminatory practice and its theories have been discredited. It is important to recognize the historical and ongoing impacts of eugenics and to actively work to combat discrimination and promote social justice.

16. secondary attack rate

The secondary attack rate (SAR) is a measure of the number of secondary cases of a disease that occur as a result of exposure to a primary case. It is calculated by dividing the number of secondary cases by the number of people exposed to the primary case, and is typically expressed as a percentage.

1. Transmission dynamics: The secondary attack rate is a measure of the transmission dynamics of a disease. It can provide insight into how easily a disease spreads within a population and can help identify the most efficient ways to control the spread of the disease.
2. Control measures: The secondary attack rate can also be used to evaluate the effectiveness of control measures, such as quarantine and isolation, in reducing the spread of a disease.
3. Comparison: The secondary attack rate can also be used to compare the transmissibility of different diseases.
4. Time frame: The secondary attack rate is typically calculated over a specific time frame, such as one week or one month, to reflect recent transmission dynamics.

5. Risk factors: The secondary attack rate can vary depending on a number of factors, including the virulence of the pathogen, the susceptibility of the population, and the effectiveness of control measures.

In summary, the secondary attack rate (SAR) is a measure of the number of secondary cases of a disease that occur as a result of exposure to a primary case. It is used to evaluate the transmission dynamics of a disease and the effectiveness of control measures, and it can provide insight into the risk factors for transmission.

17. Ice berg of disease

The iceberg of disease is a metaphor used to describe the relationship between visible and invisible forms of illness and disability. The metaphor compares visible cases of illness, represented by the tip of an iceberg, to the much larger number of invisible cases, represented by the submerged portion of the iceberg.

1. Visible illness: The visible illness represents the cases of illness that are diagnosed and treated by healthcare professionals, such as people with heart disease, cancer, and diabetes.
2. Invisible illness: The invisible illness represents the cases of illness that are not diagnosed or treated, such as people with mental health conditions, substance abuse disorders, and disability.
3. Prevalence: The metaphor illustrates that the prevalence of visible illness is much smaller than the prevalence of invisible illness.
4. Health care system: The metaphor highlights the limitations of the health care system, which tends to focus on the treatment of visible illness, while neglecting the needs of people with invisible illness.
5. Social determinants: The iceberg of disease also illustrates the importance of addressing the social determinants of health, such as poverty, education, and access to healthcare, which contribute to the development of both visible and invisible illness.
6. Public health: The iceberg of disease is an important concept in public health, as it emphasizes the need to address the full spectrum of health and illness in order to improve the overall health of a population.

In summary, the iceberg of disease is a metaphor that is used to describe the relationship between visible and invisible forms of illness and disability, and highlights the limitations of the healthcare system in addressing the needs of people with invisible illness, the importance of addressing the social determinants of health and the need for Public health to address the full spectrum of health and illness.

18. Types of screening

Screening refers to the process of identifying individuals who are at increased risk of a specific condition or disease, so that they can receive appropriate care and treatment. There are several types of screening programs, including:

1. Mass screening: Mass screening programs are designed to screen a large population for a specific condition or disease, regardless of their individual risk. This type of screening is usually done in a community setting, and may involve the use of simple, non-invasive tests, such as blood pressure and cholesterol screenings.
2. Targeted screening: Targeted screening programs are designed to screen specific subgroups of the population that are at increased risk of a specific condition or disease. This type of screening often involves a more detailed assessment and may include diagnostic tests and follow-up care.
3. Universal screening: Universal screening programs are designed to screen all individuals for a specific condition or disease. This type of screening is usually done at a specific age or at a specific point in time, and may involve the use of both mass and targeted screening methods.
4. Genetic screening: Genetic screening is the process of identifying genetic mutations that are associated with a specific condition or disease. This type of screening is usually done in individuals who have a family history of the condition or disease, or in populations that are known to be at increased risk.
5. Newborn screening: Newborn screening is the process of testing all newborns for a specific set of conditions or diseases shortly after birth. This type of screening is usually done to detect conditions that can have serious health consequences if not treated early.

19. Comprehensive mental health action plan

A comprehensive mental health action plan is a document that outlines the strategies, actions, and resources needed to improve mental health and well-being in a specific population or community. The plan is designed to address the full spectrum of mental health issues, from prevention to treatment and recovery.

1. Goals and objectives: The plan includes specific goals and objectives, such as reducing the prevalence of mental illness and suicide, improving access to mental health services, and promoting mental health and well-being.
2. Strategies: The plan includes a variety of strategies to achieve the goals and objectives, such as increasing awareness and education about mental health, improving access to mental health services, and providing support for people with mental illness and their families.
3. Implementation: The plan outlines the steps necessary for implementation, including the roles and responsibilities of different stakeholders, such as government agencies, non-governmental organizations, and the private sector.
4. Monitoring and evaluation: The plan includes a system for monitoring and evaluating the progress of the plan, to ensure that it is achieving its intended goals and objectives.
5. Resources: The plan identifies the resources that are needed to implement the plan, such as funding, personnel, and infrastructure.
6. Coordination: The plan should also include a mechanism for coordination between different stakeholders to ensure effective implementation and monitoring of the plan.

A comprehensive mental health action plan is a vital tool for improving mental health and well-being in a specific population or community. It is a roadmap for addressing the full spectrum of mental health issues, from prevention to treatment and recovery, and it is designed to ensure that all stakeholders are working together to achieve common goals.

20. Isolation and Quarantine

Isolation and quarantine are two strategies used to prevent the spread of infectious diseases.

1. Isolation: Isolation refers to the separation of individuals who are known to have a contagious disease from those who are not infected. The goal of isolation is to prevent the spread of the disease to others.
2. Quarantine: Quarantine refers to the separation and restriction of movement of individuals who have been exposed to a contagious disease but do not yet have symptoms. The goal of quarantine is to prevent the spread of the disease to others by isolating individuals before they become contagious.
3. Voluntary and mandatory: Isolation and quarantine can be either voluntary or mandatory. Voluntary isolation and quarantine is when an individual chooses to isolate or quarantine themselves, while mandatory isolation and quarantine is when an individual is ordered by a public health official to isolate or quarantine themselves.
4. Duration: The duration of isolation and quarantine varies depending on the disease and the individual's health status.
5. Monitoring: Public health officials monitor individuals in isolation and quarantine to ensure compliance and to provide support and resources as needed.
6. Legal and ethical issues: Isolation and quarantine can raise legal and ethical issues, such as the right to privacy and autonomy, the use of force, and the potential impact on vulnerable populations.

In summary, isolation and quarantine are two strategies used to prevent the spread of infectious diseases. Isolation is used for individuals who are known to have a contagious disease, while quarantine is used for individuals who have been exposed but do not yet have symptoms. Both can be either voluntary or mandatory and raises legal and ethical issues.
