# Supplementary material for: Performance of ChatGPT on the India Undergraduate Community Medicine Examination: Cross-Sectional Study
Source: JMIR Form Res. 2024 Mar 25;8:e49964. doi: 10.2196/49964 (PMC11002731; doi:10.2196/49964)
Supplement: Multimedia Appendix 3 [file formative_v8i1e49964_app3.pdf]

Multimedia Appendix 3: ChatGPT answers for the questions in Paper II

Section 1 Long essay

1. Define infant mortality rate (IMR). Describe in detail the factors for IMR and preventive and control measures.

Infant mortality rate (IMR) is a key indicator of the overall health and well-being of a population. It is defined as the number of deaths of infants under one year of age per 1,000 live births. IMR is considered a sensitive indicator of the health status of a community as it reflects the impact of both genetic and environmental factors on the survival of infants. In recent years, IMR has been used to monitor the progress of countries in achieving the United Nations' Millennium Development Goal 4, which aims to reduce the under-five mortality rate by two-thirds between 1990 and 2015.

There are several factors that contribute to high IMR. Some of the major ones include:

- poor maternal health and nutrition: pregnant women who are malnourished or have poor health are more likely to have low birth weight babies, which are at a higher risk of dying in the first year of life.
- inadequate prenatal care: lack of access to prenatal care increases the risk of maternal and fetal complications, which can lead to infant death.
- lack of access to safe and affordable healthcare: infants born in areas without access to basic healthcare services are at a higher risk of dying from preventable causes such as infections and diarrhea.
- poor sanitation and hygiene: lack of access to clean water and adequate sanitation facilities increases the risk of infectious diseases, which can lead to infant death.
- high rates of infectious diseases: malaria and diarrhea are major causes of infant death in developing countries.
- socio-economic and demographic factors: poverty and low education levels are strongly associated with high IMR.

Preventive and control measures to reduce IMR include:

- improving maternal health and nutrition: this can be achieved through programs that provide pregnant women with access to nutritious food, supplements, and prenatal care.
- increasing access to prenatal care and safe delivery services: this can be achieved through programs that provide pregnant women with access to trained healthcare providers and facilities for delivery.
- increasing access to basic healthcare services: this can be achieved through programs that provide infants with access to vaccination and treatment for infectious diseases.
- promoting good sanitation and hygiene practices: this can be achieved through programs that provide communities with access to clean water and adequate sanitation facilities.

- providing clean water and adequate sanitation facilities: this can be achieved through programs that provide communities with access to clean water and adequate sanitation facilities.
- implementing programs to reduce poverty and improve education: this can be achieved through programs that provide families with access to income-generating activities and educational opportunities.
- promoting family planning to reduce high birth rates: this can be achieved through programs that provide couples with access to family planning services and information.

It also includes community-based interventions for maternal and child health, including promotion of breast-feeding, immunization, oral rehydration therapy, and essential newborn care. These interventions are known to be effective in reducing IMR, and should be implemented in parallel with other IMR reduction strategies.

Additionally, monitoring the IMR at regular intervals, and evaluating the impact of interventions implemented to reduce IMR and taking corrective measures in case of lack of progress. This can be achieved through programs that provide communities with access to data on IMR and other indicators of maternal and child health.

In conclusion, reducing IMR requires a multifaceted approach that addresses the underlying social, economic, and environmental factors that contribute to poor health outcomes in infants and children. Programs and policies that target these factors, and that provide access to basic healthcare services, clean water and sanitation, and maternal and child health services, are the most effective in reducing IMR. Monitoring the progress of these interventions, and implementing corrective measures in case of lack of progress, is crucial to achieving the

2. Describe in detail epidemiology of TB & write in detail about prevention, control & NTEP (India).

Tuberculosis (TB) is a contagious bacterial infection that primarily affects the lungs, but can also affect other parts of the body. It is caused by Mycobacterium tuberculosis, and is spread through the air when an infected person coughs, sneezes, or talks. TB is one of the world's leading causes of death from a single infectious agent, and is a major public health problem, particularly in developing countries.

Epidemiology of TB:

- Globally, TB is a major public health problem. In 2019, an estimated 10 million people developed TB and 1.4 million died from the disease.
- India has the highest burden of TB in the world, accounting for 27% of global TB cases and 20% of global TB deaths.
- The majority of TB cases in India occur in adults aged 15-59 years.
- The incidence of TB in India is highest in the age group of 15-44 years.
- The incidence of TB is also higher in males than females.

- TB disproportionately affects marginalized populations, such as people living in poverty, people living with HIV, and people who use tobacco.
- Multi-drug-resistant TB (MDR-TB) is a growing problem in India, with an estimated 3.9% of TB cases being MDR-TB.

Prevention, control and NTEP (India):

- The National TB Control Programme (NTBCP) was launched in 1962 in India, and was later renamed as the Revised National TB Control Programme (RNTCP) in 1997.
- The RNTCP is based on the World Health Organization (WHO) recommended DOTS (Directly Observed Treatment, Short-course) strategy, which aims to provide standardized, high-quality TB care and treatment to all TB patients.
- The RNTCP focuses on early case detection, standardized treatment, and monitoring of treatment outcomes.
- The program uses a decentralized approach, with TB care and treatment provided at the primary healthcare level, with active case finding through intensive contact tracing and screening of high-risk populations.
- The program also provides free TB drugs, and provides financial and other incentives to patients to ensure completion of treatment.
- The program has made significant progress in reducing TB incidence and mortality in India. From 2000 to 2019, TB incidence fell by 27% and TB deaths fell by 42%.
- However, the program faces several challenges, including inadequate funding, limited human resources, and inadequate infrastructure.
- The RNTCP also has a component for MDR-TB management, which includes providing free second-line drugs, and providing DOTS-Plus (an intensified version of DOTS) for the management of MDR-TB.
- The program has made progress in the management of MDR-TB, with the percentage of patients successfully treated increasing from 42% in 2007 to 60% in 2016.
- The program also includes various other components like TB-HIV collaborative activities, TB control in private sector, and TB research.

In addition to the RNTCP, there are also other efforts underway to control TB in India, such as:

- The National Strategic Plan for Tuberculosis Elimination (2017-2025) aims to achieve the WHO's End TB strategy targets by 2025.
- The TB Free India campaign, launched in 2018, aims to mobilize political and social commitment to end TB, and to increase investment in TB control.
- The program also includes various other components like TB-HIV collaborative activities, TB control in private sector, and TB research.

In conclusion, TB is a major public health problem in India, and the RNTCP has made significant progress in reducing TB incidence and mortality

Section 2 Short essay

3. Elements of primary health care

Primary health care (PHC) is an approach to health care that focuses on providing comprehensive and accessible health care services to individuals and communities, with an emphasis on preventing illness and promoting health. The World Health Organization (WHO) defines primary health care as "essential health care based on practical, scientifically sound and socially acceptable methods and technology made universally accessible to individuals and families in the community through their full participation and at a cost that the community and the country can afford."

Elements of primary health care:

1. Community participation: Community participation is a key element of PHC, as it helps to ensure that health services are responsive to the needs and preferences of the community, and that the community is engaged in the planning, implementation, and evaluation of health services.
2. Inter-sectoral collaboration: PHC requires collaboration and coordination between different sectors, such as health, education, housing, and environment, to address the social determinants of health.
3. Health promotion: Health promotion is an important element of PHC, as it focuses on empowering individuals and communities to take control of their own health, and to make healthy choices.
4. Disease prevention: PHC places a strong emphasis on disease prevention, through activities such as vaccination, health education, and screening programs.
5. Accessible and affordable health services: PHC aims to make health services accessible and affordable to all individuals and communities, regardless of their social or economic status.
6. Integrated care: PHC is an integrated approach to care that addresses the health needs of individuals and communities as a whole, rather than focusing on specific diseases or conditions.
7. Health workforce: PHC requires a well-trained and motivated health workforce, which includes a range of different health professionals, such as doctors, nurses, and community health workers.
8. Technology: PHC uses appropriate technology, which is cost-effective, affordable, and easy to use, to deliver health services.
9. Monitoring and evaluation: PHC requires ongoing monitoring and evaluation of health services, to ensure that they are effective and responsive to the needs of the community.
10. Health systems: PHC requires strong health systems that are able to deliver quality health services to all individuals and communities in an equitable and sustainable manner.

In conclusion, primary health care is an approach to health care that focuses on providing comprehensive and accessible health care services to individuals and communities, with an emphasis on preventing illness and promoting health. It is an integrated approach that addresses the health needs of individuals and communities as a whole, rather than focusing on specific diseases or conditions. It requires the active participation of communities and collaboration between different sectors, and requires a well-trained and motivated health workforce, appropriate technology and strong health systems.

4. Planning cycle

The planning cycle is a process used to identify and address specific issues, goals or objectives, and to develop a plan for achieving them. The cycle typically includes several key stages, including assessment, planning, implementation, and evaluation.

1. Assessment: The first stage of the planning cycle is assessment. This stage involves identifying the problem or issue that needs to be addressed, as well as gathering data and information that will be used to develop a plan. The assessment stage may include conducting a needs assessment, gathering data on the current situation, and identifying the stakeholders who will be involved in the planning process.
2. Planning: The second stage of the planning cycle is planning. This stage involves developing a plan to address the problem or issue identified in the assessment stage. The plan should include specific, measurable goals or objectives, as well as strategies and tactics for achieving them. Planning may also involve identifying resources, such as funding or staff, that will be needed to implement the plan.
3. Implementation: The third stage of the planning cycle is implementation. This stage involves taking the plan developed in the planning stage and putting it into action. Implementation may include activities such as training staff, developing policies and procedures, and launching a public awareness campaign.
4. Evaluation: The final stage of the planning cycle is evaluation. This stage involves assessing the effectiveness of the plan and making any necessary adjustments. Evaluation may include analyzing data to determine whether the goals or objectives have been met, and identifying any areas where the plan can be improved.
5. Continual improvement: Continual improvement is an important aspect of the planning cycle as it helps to ensure that the plan remains relevant and effective over time. Continual improvement may involve adjusting goals or objectives, revising the plan, or making changes to the way the plan is implemented.

It is an iterative process, and after the evaluation stage, the cycle starts again with assessment, then planning, implementation and evaluation. This allows organizations to continuously improve their plans and strategies over time, which is important as the environment and conditions are constantly changing.

In conclusion, the planning cycle is an important process that is used to identify and address specific issues, goals or objectives and to develop a plan for achieving them. It includes several key stages, including assessment, planning, implementation, and evaluation, and is an iterative process that allows organizations to continuously improve their plans and strategies over time.

5.Syndromic approach of STDs

The syndromic approach to the management of sexually transmitted infections (STIs) is a method of identifying and treating patients based on the presence of symptoms, rather than laboratory testing. This approach is particularly useful in settings where laboratory testing may be unavailable or cost-prohibitive.

The syndromic approach is based on the recognition of the common signs and symptoms of STIs, such as genital ulcers, vaginal discharge, and lower abdominal pain. Patients presenting with these symptoms are then treated with a standardized treatment regimen, regardless of the specific STI that may be causing the symptoms.

One of the main advantages of the syndromic approach is its simplicity. It requires minimal diagnostic equipment, making it well-suited for resource-limited settings. Additionally, the syndromic approach allows for prompt treatment of patients, which can reduce the risk of transmission and the development of complications.

However, the syndromic approach does have some limitations. Since it does not rely on laboratory testing, it may lead to over-treatment of patients with no STI, or under-treatment of patients with an STI that is not causing symptoms. Furthermore, it is not able to identify asymptomatic patients with STIs, which can lead to ongoing transmission.

In order to overcome these limitations, the World Health Organization (WHO) recommends a combination of the syndromic and laboratory-based approaches for the management of STIs. This approach involves providing syndromic treatment to patients with symptoms, while also conducting laboratory testing to confirm the diagnosis and identify asymptomatic cases.

In addition, the WHO recommends a combination of the syndromic approach with the use of point-of-care diagnostic tests. Point-of-care diagnostic tests are rapid diagnostic tests that can be performed at the same location as the patient consultation, which eliminates the need for laboratory testing and reduces the time to treatment.

In conclusion, the syndromic approach to the management of STIs is a useful method for identifying and treating patients in settings where laboratory testing may be unavailable or cost-prohibitive. However, it does have some limitations, which can be overcome by using a combination of the syndromic and laboratory-based approaches, and by using point-of-care diagnostic tests. This approach allows for prompt treatment of patients, reduces the risk of transmission, and identifies asymptomatic patients with STIs.

6.Models of health education

Health education is the process of providing information and skills to individuals, communities and populations in order to improve their health and well-being. There are several different models of health education that have been developed to guide this process. These models differ in terms of their focus, goals and strategies, but they all share the common aim of improving health outcomes.

One of the most widely used models of health education is the Health Belief Model (HBM). This model suggests that individuals' health behaviors are determined by their perceptions of the susceptibility of a health threat, the severity of the threat, the benefits of taking action, and the barriers to taking action. This model is often used to guide interventions aimed at changing health behaviors by addressing these perceptions.

Another model of health education is the Transtheoretical Model (TTM), also known as the Stages of Change Model. This model suggests that individuals progress through different stages of readiness to change their health behaviors. The stages include precontemplation, contemplation, preparation, action and maintenance. The TTM is often used to guide interventions aimed at helping individuals move through these stages and make lasting changes to their health behaviors.

The Social Cognitive Theory (SCT) is another model of health education which emphasizes the role of social and environmental factors in shaping health behaviors. The SCT suggests that individuals learn and adopt health behaviors by observing and imitating the behaviors of others, and that these behaviors are shaped by the social and physical context in which they occur. This model is often used to guide interventions aimed at changing health behaviors by addressing the social and environmental factors that influence them.

The Community-based participatory research (CBPR) is another model of health education which focuses on community involvement in the research process. This model emphasizes the importance of involving community members in the identification of health problems, the development of interventions and the evaluation of outcomes. This approach is often used in addressing health disparities and promoting health equity.

Finally, the Ecological Model of Health Promotion is a model that considers the impact of multiple levels of influence on health, including individual, interpersonal, organizational, community, and policy levels. This model is often used to guide interventions aimed at addressing the complex and interrelated factors that influence health outcomes.

In conclusion, models of health education provide a framework for understanding and addressing the factors that influence health behaviors. These models have different focuses, goals, and strategies, but they all share the common aim of improving health outcomes. By understanding the different models of health education and how they can be applied, practitioners can develop more effective and targeted interventions to improve the health and well-being of individuals, communities, and populations.

7.Epidemiology of RTA

Road traffic accidents (RTA) are a major public health concern worldwide, and are a leading cause of morbidity and mortality. Epidemiology is the study of the distribution and determinants of health-related states or events, including RTA. Understanding the epidemiology of RTA is essential for developing effective interventions to reduce the burden of these accidents.

The World Health Organization (WHO) estimates that road traffic injuries are the leading cause of death among people aged 15-29 years, and that they are responsible for 1.35 million deaths globally each year. This number is projected to increase to 1.9 million deaths by 2020, unless effective interventions are implemented.

The epidemiology of RTA varies across countries and regions, and is influenced by a number of factors, including population density, urbanization, economic development, and road

infrastructure. In low- and middle-income countries, RTA are more common in rural areas, where infrastructure is often poor, and vehicles are older and less well-maintained. In high-income countries, RTA are more common in urban areas, where population density is high and traffic is more congested.

In addition to geographic variations, there are also demographic variations in the epidemiology of RTA. Young males are at a higher risk of being involved in RTA than other groups, and this risk increases with age. This is due in part to risk-taking behavior and lack of experience, but also to a lack of access to safe transportation options.

Risk factors for RTA include a range of individual and environmental factors, including speed, alcohol and drug use, fatigue, and distraction. Traffic laws and enforcement, road infrastructure, and vehicle safety standards also play a role in determining the incidence of RTA.

In terms of prevention, effective interventions include reducing speed limits, increasing enforcement of traffic laws, and promoting the use of seat belts, helmets, and other safety equipment. Improving road infrastructure, such as by building sidewalks and bike lanes, can also reduce the risk of RTA. Additionally, public awareness campaigns and educational programs can help to change behavior and reduce the risk of RTA.

In conclusion, the epidemiology of RTA is a complex issue that is influenced by a variety of factors, including geographic, demographic, and environmental factors. Understanding the epidemiology of RTA is essential for developing effective interventions to reduce the burden of these accidents. Interventions should be tailored to the specific context and target population, and focus on reducing risk factors, improving road infrastructure, and changing behavior.

8. occupational cancers

Occupational cancers refer to cancers that occur as a result of exposure to cancer-causing agents in the workplace. These cancers are caused by a variety of chemicals, physical agents, and biological agents that are present in the workplace environment. They are a significant public health concern, as they account for a significant proportion of all cancer deaths worldwide.

Epidemiology studies have shown that certain occupations have a higher risk of certain types of cancer. For example, lung cancer is more common among workers who are exposed to asbestos, radon, and other lung carcinogens, such as those in the construction, mining and shipyard industries. Similarly, bladder cancer is more common among workers who are exposed to certain chemicals, such as those used in the rubber, chemical, and dye industries.

Occupational cancers can also occur due to exposure to physical agents such as ionizing radiation, ultraviolet radiation, and electromagnetic fields. For example, skin cancer is more common among workers who are exposed to UV radiation, such as those in the agriculture, construction and beauty industries.

The risk of occupational cancer depends on the type and level of exposure to the carcinogenic agent, as well as other factors such as age, gender, and overall health status. The risk also depends on the duration of exposure, as well as the time between exposure and the onset of cancer.

Preventing occupational cancers requires a multi-faceted approach. The first step is to identify and assess the risk of exposure to cancer-causing agents in the workplace. This can be done through workplace monitoring and surveillance programs, as well as by conducting health assessments of workers.

Once the risk of exposure has been identified, appropriate control measures can be implemented to reduce or eliminate the exposure. These measures can include engineering controls, such as ventilation systems, to reduce exposure to carcinogens, as well as administrative controls, such as work practices and procedures, to reduce exposure to physical agents.

Personal protective equipment (PPE) can also be used to reduce exposure to cancer-causing agents. For example, the use of respirators can help to reduce exposure to lung carcinogens, while protective clothing can help to reduce exposure to skin carcinogens.

Education and training are also important components of preventing occupational cancers. Workers should be informed about the risks associated with exposure to cancer-causing agents, and should be trained on how to use PPE and other control measures effectively.

In conclusion, occupational cancers are a significant public health concern, accounting for a significant proportion of all cancer deaths worldwide. The risk of occupational cancer depends on the type and level of exposure to the carcinogenic agent, as well as other factors such as age, gender, and overall health status. To prevent occupational cancers, a multi-faceted approach is needed that includes identifying and assessing the risk of exposure, implementing appropriate control measures, using PPE, and providing education and training.

9. Oral contraceptives

Oral contraceptives, also known as birth control pills, are medications that are taken orally to prevent pregnancy. They work by preventing ovulation, or the release of an egg from the ovary. Oral contraceptives are a popular and effective form of birth control, and have been used by women for over 50 years.

There are two types of oral contraceptives: combined and progestin-only. Combined oral contraceptives contain both an estrogen and a progestin, while progestin-only pills contain only a progestin. Both types of pills work to prevent pregnancy by thickening the mucus in the cervix, which makes it difficult for sperm to reach and fertilize an egg.

The effectiveness of oral contraceptives depends on a number of factors, including how well the pills are taken, and whether or not the woman is taking any other medications that may

interact with the pills. When used correctly and consistently, the combined pill is 99% effective at preventing pregnancy, and the progestin-only pill is 94% effective.

One of the major benefits of oral contraceptives is that they can help regulate a woman's menstrual cycle, making it more predictable and reducing the severity of cramps and other symptoms. They can also reduce the risk of certain types of cancer, such as ovarian and endometrial cancer.

However, oral contraceptives do have some potential side effects, such as nausea, headaches, and changes in mood. These side effects usually go away after a few months of use. Additionally, there is a small risk of blood clots, which can be serious or even life-threatening.

It is also important to note that oral contraceptives do not protect against sexually transmitted infections (STIs), and it is recommended that condoms be used in addition to oral contraceptives for protection against STIs.

In conclusion, oral contraceptives, also known as birth control pills, are a popular and effective form of birth control that have been used by women for over 50 years. They work by preventing ovulation, and when used correctly and consistently, can be 99% effective at preventing pregnancy. They have many benefits, including regulating menstrual cycles and reducing the risk of certain types of cancer. However, they do have potential side effects and do not protect against STIs. It is important to consult with a healthcare provider to determine if oral contraceptives are the right choice for an individual, and to discuss any potential risks or side effects.

10. Ford Foundation

The Ford Foundation is a private foundation that was established in 1936 by Edsel Ford, the son of Henry Ford, and his wife, Eleanor Clay Ford. The foundation is based in New York City and is one of the oldest, largest, and most influential foundations in the United States.

The mission of the Ford Foundation is to reduce poverty and injustice, promote democratic values, and advance human welfare. To achieve this mission, the foundation focuses on several key areas, including:

1. Economic Opportunity and Assets: The foundation works to promote economic opportunity and build assets for low-income individuals and communities. This includes support for microfinance programs, community development financial institutions, and other initiatives that help low-income people access credit, savings, and other financial services.
2. Education: The Ford Foundation works to improve access to quality education for all students, with a particular focus on disadvantaged students and students from marginalized communities. This includes support for educational programs and initiatives that help students succeed in school, improve their academic skills, and prepare for college and careers.

3. Gender, Racial, and Ethnic Justice: The foundation works to promote gender, racial, and ethnic justice by supporting organizations and initiatives that address discrimination and inequality, and promote the rights and well-being of marginalized and underrepresented groups.
4. Human Rights: The Ford Foundation works to promote human rights by supporting organizations and initiatives that defend the rights of marginalized and oppressed people, and work to end discrimination and injustice.
5. Creativity and Free Expression: The foundation also supports organizations and initiatives that promote creativity and free expression, such as the arts, independent media, and free speech.

The Ford Foundation has a global reach, with programs and initiatives in more than 60 countries around the world. The foundation has invested more than $17 billion in grants and programs since it was established, and it continues to be a major philanthropic force in the United States and around the world.

In conclusion, The Ford Foundation is a private foundation that was established in 1936 by Edsel Ford, the son of Henry Ford, and his wife, Eleanor Clay Ford. The foundation's mission is to reduce poverty and injustice, promote democratic values, and advance human welfare. The foundation focuses on several key areas including economic opportunity, education, gender, racial, and ethnic justice, human rights, and creativity and free expression. The Ford Foundation has a global reach, with programs and initiatives in more than 60 countries around the world, and it continues to be a major philanthropic force in the United States and around the world.

Section 3: Short answers

11. pneumococcal vaccine

Pneumococcal vaccine is a vaccine that is used to protect against infections caused by the bacterium Streptococcus pneumoniae. This bacterium is responsible for a wide range of illnesses, including pneumonia, meningitis, and sepsis. Pneumococcal vaccine is recommended for certain groups of people who are at high risk of developing serious complications from pneumococcal infections, including infants, children, older adults, and people with certain underlying health conditions.

There are two types of pneumococcal vaccines available: the pneumococcal conjugate vaccine (PCV) and the pneumococcal polysaccharide vaccine (PPSV). The PCV is recommended for infants and children, and is given in a series of doses starting at 2 months of age. The PPSV is recommended for adults, particularly those who are 65 years or older, and those with certain underlying health conditions.

The pneumococcal vaccine works by exposing the body to a small amount of the bacteria, which triggers the immune system to produce antibodies against the bacteria. These antibodies help protect the person from getting infected with the bacteria in the future. The

vaccine is generally considered safe and well-tolerated, with side effects being generally mild and short-lived.

The vaccine is highly effective in preventing pneumococcal infections, and it has been shown to reduce the incidence of pneumonia, meningitis, and sepsis caused by S. pneumoniae. It also helps to reduce the spread of the bacteria to others, and has been shown to be particularly effective in preventing serious complications and death from pneumococcal infections.

In conclusion, Pneumococcal vaccine is a vaccine that is used to protect against infections caused by the bacterium Streptococcus pneumoniae. There are two types of pneumococcal vaccines available: the pneumococcal conjugate vaccine (PCV) and the pneumococcal polysaccharide vaccine (PPSV). The PCV is recommended for infants and children, and the PPSV is recommended for adults, particularly those who are 65 years or older, and those with certain underlying health conditions. The vaccine is generally considered safe and well-tolerated, with side effects being generally mild and short-lived. It is highly effective in preventing pneumococcal infections, and it helps to reduce the spread of the bacteria to others, and has been shown to be particularly effective in preventing serious complications and death from pneumococcal infections.

12. Plumbism

Plumbism, also known as lead poisoning, is a condition that occurs when a person is exposed to excessive amounts of lead. Lead is a toxic metal that is commonly found in the environment, and exposure to it can occur through a variety of sources, including contaminated water, lead-based paint, and lead-contaminated dust and soil.

Symptoms of plumbism can vary depending on the level of exposure and can include abdominal pain, constipation, fatigue, headaches, irritability, and memory loss. In severe cases, plumbism can cause anemia, kidney damage, and neurological damage, including seizures and coma.

Children are particularly vulnerable to lead exposure, and plumbism can cause developmental delays and behavioral problems in kids. Pregnant women are also at risk, as lead exposure can harm the developing fetus.

There are several ways to prevent plumbism. The most important step is to identify and eliminate sources of lead exposure in the home, such as lead-based paint and lead-contaminated drinking water. This can be done by testing for lead in water and paint, and by removing or covering lead-based paint. Other measures that can help prevent plumbism include regular hand washing, especially before eating, and regular cleaning of floors and window sills to remove lead dust.

It is also important to get regular blood lead level testing, particularly if you are at high risk of lead exposure. If a person is diagnosed with plumbism, treatment will typically involve removing the person from the source of lead exposure and chelating therapy, which is a medication that binds to lead and helps remove it from the body.

In summary, Plumbism is a condition that occurs when a person is exposed to excessive amounts of lead, which is a toxic metal that is commonly found in the environment. Symptoms of plumbism can vary depending on the level of exposure and can include abdominal pain, constipation, fatigue, headaches, irritability, and memory loss. Children are particularly vulnerable to lead exposure, and plumbism can cause developmental delays and behavioral problems in kids. Pregnant women are also at risk, as lead exposure can harm the developing fetus. The most important step is to identify and eliminate sources of lead exposure in the home, such as lead-based paint and lead-contaminated drinking water and chelating therapy is the main treatment for plumbism.

13. Network analysis

Network analysis is a method used to study the relationships and interactions between individuals or groups within a particular system. It is a tool that can be used to understand the structure and dynamics of social networks, communication networks, transportation networks, and many other types of systems.

Network analysis is used to identify key players or actors within a network, as well as the patterns of

Short essay (300 words) on network analysis in health planning and management

Network analysis is a powerful tool that can be used in health planning and management to understand the structure and dynamics of health systems. It can be used to identify key actors and organizations within a health system, as well as the relationships and interactions between them.

One common application of network analysis in health is to study the spread of infectious diseases within a population. By mapping the connections between individuals and the flow of pathogens through a network, researchers can identify key transmission pathways and high-risk groups, and design targeted interventions to interrupt the spread of the disease.

Another application of network analysis in health is to study the relationships and interactions between different healthcare providers and organizations. By identifying key actors and clusters within a healthcare system, researchers can identify areas of strength and weakness and design interventions to improve the overall performance and efficiency of the system.

Network analysis can also be used in the management of chronic diseases by identifying the patients and the healthcare providers who are the most influential in their care. This can help to improve patient outcomes by targeting interventions at the most critical nodes within the network.

Overall, network analysis is a valuable tool for understanding and improving the structure and function of health systems. It can be used to identify key actors and relationships, target interventions, and optimize the performance of health systems at all levels.

14. Road to Health chart

The Road to Health chart, also known as the "growth chart," is a tool used to track a child's growth and development over time. It is a visual representation of a child's weight, height, and head circumference measurements taken at regular intervals. The chart is used to monitor a child's growth and detect any potential issues or deviations from the norm.

The Road to Health chart is divided into different age ranges, such as birth to 2 years, 2 to 5 years, and so on. Each age range has specific growth benchmarks for weight, height, and head circumference, which are based on the World Health Organization's (WHO) Child Growth Standards. These benchmarks provide a reference for what is considered normal growth for a child of a particular age and gender.

Healthcare providers use the Road to Health chart to monitor a child's growth and development during routine check-ups and vaccinations. They plot the child's measurements on the chart, and can then compare them to the growth benchmarks for that age range. If a child's measurements fall outside of the benchmark range, it may indicate a potential growth problem or underlying health issue.

The Road to Health chart is also used to track a child's progress over time, and to identify any patterns or trends in their growth. This information can be used to adjust a child's diet, provide additional support and resources, or refer the child to a specialist if necessary.

Overall, the Road to Health chart is a valuable tool for monitoring a child's growth and development, identifying potential issues, and ensuring that children are receiving the support and resources they need to grow and thrive.

15. Juvenile deliquency

Juvenile delinquency refers to the criminal behavior of minors, typically those under the age of 18. Juvenile delinquency can take many forms, including truancy, vandalism, drug use, and violent crime.

There are a number of factors that can contribute to juvenile delinquency. Family dynamics, such as poverty, abuse, or neglect, can play a significant role. Social and economic factors, such as unemployment, poverty, and poor education, can also contribute to delinquent behavior. Additionally, individual factors such as poor mental health, substance abuse, and a lack of positive role models can lead to juvenile delinquency.

Preventing juvenile delinquency is a complex task that requires a multi-faceted approach. One important strategy is to provide young people with positive role models and opportunities for constructive activities, such as sports, art, music, and volunteer work. This can help to build self-esteem, teach discipline, and provide a sense of purpose.

Another important strategy is to improve access to education and job training. This can help to reduce poverty and provide young people with the skills they need to succeed in the workforce.

Parenting education and support is also an important strategy in preventing juvenile delinquency. Parents play a crucial role in shaping their children's behavior, and providing them with the knowledge, skills, and support they need to raise healthy, well-adjusted children can help to reduce the risk of delinquent behavior.

In addition, providing mental health services and substance abuse treatment can help to address underlying issues that may be contributing to delinquent behavior.

Overall, preventing juvenile delinquency requires a collaborative effort from parents, educators, law enforcement, community organizations, and government agencies. By working together and addressing the underlying issues that contribute to delinquent behavior, we can help to reduce the incidence of juvenile delinquency and create safer, healthier communities for our young people.

16. Post exposure prophylaxis of rabies

Post-exposure prophylaxis (PEP) is a set of medical treatments given after a person has been exposed to a potentially infected animal or person in order to prevent the development of rabies. The goal of PEP is to prevent the virus from infecting the person and to prevent the onset of symptoms.

The first step in PEP is to thoroughly clean the wound with soap and water to remove any virus present on the skin. This should be followed by the administration of a rabies immune globulin, which contains antibodies that can neutralize the virus. The immune globulin is usually given in the muscle near the wound, as well as in the wound itself.

The second step of PEP is to administer a series of rabies vaccine doses. This is usually done in the deltoid muscle of the arm and usually four or five doses are given over a period of 28 days. The vaccine is used to stimulate the immune system to produce its own antibodies against the rabies virus.

It is important to seek medical attention as soon as possible after exposure to rabies. The earlier PEP is started, the more effective it is in preventing the development of rabies. In addition, PEP is most effective when given as soon as possible after exposure.

PEP is also recommended for certain high-risk groups, such as laboratory workers who handle the rabies virus, veterinarians, and animal control workers. In some cases, PEP may also be recommended for people who have been bitten or scratched by a wild animal or a domestic animal that is suspected of having rabies.

In summary, PEP is a set of medical treatments given to prevent the development of rabies after exposure to the virus. It is important to seek medical attention as soon as possible after

exposure and to follow the recommended PEP regimen. PEP can be effective in preventing rabies if given in a timely manner.

17. Barriers of communication

Barriers of communication refer to any obstacles or hindrances that impede the successful transfer of information or message between a sender and a receiver. These barriers can occur at any stage of the communication process, including encoding, transmission, decoding, or feedback.

Some common barriers of communication include:

1. Language barriers: When the sender and receiver do not speak the same language or use different jargon, it can be difficult for the message to be understood.
2. Perceptual barriers: Different people perceive things differently, and this can lead to misunderstandings or misinterpretations of the message.
3. Physical barriers: Poor lighting, noise, distance, and other physical factors can make it difficult for the message to be received.
4. Emotional barriers: Negative emotions such as anger, fear, or anxiety can impair communication.
5. Cultural barriers: Different cultures have different communication styles and norms, which can lead to misunderstandings or misinterpretations of the message.
6. Semantic barriers: Words may have different meanings to different people, or people may use the same words to mean different things.
7. Personal barriers: Personal biases, prejudices, or lack of interest in the message can lead to communication barriers.

Overcoming these barriers involves identifying them and taking steps to address them, such as using plain language, repeating the message, providing feedback, and ensuring that the message is clear and relevant to the receiver.

18. Provision of services under ICDS

The Integrated Child Development Services (ICDS) is a government of India program that aims to improve the health and nutrition of children under six years of age, and pregnant and lactating women. The program provides a range of services through a network of Anganwadi centres, which are community-based centres that provide health and nutrition services.

The services provided under ICDS include:

1. Supplementary nutrition: This includes providing hot, cooked meals or take-home rations to children under six years of age, pregnant and lactating women. The meals are designed to provide the necessary nutrients to support growth and development.
2. Immunization: Children are provided with vaccinations to protect against a range of diseases, such as measles, polio, and diphtheria.

3. Health check-ups: Children are provided with regular health check-ups to identify any health problems early on and to ensure that they are receiving the necessary medical care.
4. Referral services: Children and women with health problems are referred to appropriate health facilities for further treatment.
5. Pre-school education: Children are provided with pre-school education to support their cognitive development and to prepare them for primary school.
6. Nutrition and health education: Women are provided with education on nutrition, child care, and maternal health to support them in caring for their children.
7. Non-formal education: Non-formal education services are provided to children and adults who have missed out on formal education.
8. counseling and referral services for adolescent girls.

The ICDS program is implemented through a network of Anganwadi centres, which are staffed by trained Anganwadi workers who provide the services. The program also involves the participation of the community, who are involved in the planning and implementation of the program.

In this way ICDS is a very important program in India to improve the health and nutrition of children and women, it's services are provided by the government and community based centers and is staffed by trained workers. This program plays an important role in the overall development of the country.

19. DASH diet

The DASH diet, which stands for "Dietary Approaches to Stop Hypertension," is a dietary pattern that emphasizes the consumption of fruits, vegetables, whole grains, low-fat dairy products, and lean protein sources. It also limits the intake of sodium, saturated fat, and added sugars. The DASH diet was developed by the National Heart, Lung, and Blood Institute (NHLBI) as a way to lower blood pressure and reduce the risk of heart disease, stroke, and kidney disease.

The DASH diet is rich in nutrients that are known to lower blood pressure, such as potassium, magnesium, and calcium. It also includes a moderate amount of protein and a limited amount of saturated fat. The diet encourages the consumption of fruits, vegetables, and whole grains, which are high in fiber and help to reduce cholesterol levels.

The DASH diet is a healthy eating plan that can be easily incorporated into an individual's lifestyle. It does not require restrictive measures such as counting calories or eliminating entire food groups, and it can be adapted to different cultural and economic situations. The DASH diet can be followed by people of all ages, including children, and it is suitable for vegetarians and vegans.

To follow the DASH diet, individuals should aim to eat a variety of foods from all food groups and to limit their intake of sodium, saturated fat, and added sugars. The NHLBI recommends that individuals aim to consume 2,000 calories per day and to limit their intake of sodium to 2,300 milligrams or less per day.

In conclusion, the DASH diet is a healthy eating pattern that emphasizes the consumption of fruits, vegetables, whole grains, low-fat dairy products, and lean protein sources. It limits the intake of sodium, saturated fat, and added sugars. The DASH diet can help lower blood pressure and reduce the risk of heart disease, stroke, and kidney disease.

20. GOBI

The GOBI (Growth and Opportunities for Boys and Infants) initiative is a program that aims to improve the health and well-being of young children, particularly boys, in developing countries. The initiative focuses on three main areas: growth promotion, which includes providing nutrition education and supplements to mothers and young children; immunization, which includes increasing access to vaccines and promoting vaccination coverage; and disease control, which includes providing treatment and care for common childhood illnesses such as diarrhea, pneumonia, and malaria. The program also aims to empower and educate mothers and communities to improve the overall health and well-being of children. The GOBI initiative is implemented through a variety of channels, including community-based programs, health facilities, and partnerships with other organizations and governments. The goal of the initiative is to reduce child mortality and improve the overall health and well-being of children in developing countries.
